# Green tea (*Camellia sinensis* (L.) Kuntze) nanoemulgel promotes buccal mucosal ulcer repair

**DOI:** 10.1016/j.jtumed.2026.04.004

**Published:** 2026-05-12

**Authors:** Juni Handajani, Regina T.C. Tandelilin, Heriati Sitosari, Rezmelia Sari, Dyah Irnawati, Puteri Shafinaz Abdul-Rahman, Intan D. Septiani, Nafisa A. Puspa

**Affiliations:** a Department of Oral Biology, Faculty of Dentistry, Universitas Gadjah Mada, Indonesia; b Department of Periodontology, Faculty of Dentistry, Universitas Gadjah Mada, Indonesia; c Department of Dental Biomaterial, Faculty of Dentistry, Universitas Gadjah Mada, Indonesia; d Department of Molecular Medicine, Faculty of Medicine, Universiti Malaya, Malaysia; e Student of Master Dental Science Study Program, Faculty of Dentistry, Universitas Gadjah Mada, Indonesia

**Keywords:** نانوإيمُلجل مستخلص الشاي الأخضر, القرح الرضّية في مخاطية الخد, التروية الدموية, تعبير إنزيم الأكسدة الحلقية-2, التفاعل التحسسي, الهدف الثالث من أهداف التنمية المستدامة: الصحة الجيدة والرفاه, Allergic reaction, COX-2 expression, Nanoemulgel green tea (*Camellia sinensis*) extract, Perfusion, SDG 3 (Good Health and Well Being), Wound traumatic buccal mucosal ulcers

## Abstract

The wound healing process involves various cellular and molecular mechanisms. Traumatic ulcers are lesions on the oral mucosa caused by injury. A nanoemulgel made from catechin-rich green tea extract is being developed as a potential method for accelerating the healing of oral wounds.

**Objectives:**

The aim was to evaluate whether a green tea (*Camellia sinensis* (L.) Kuntze) extract nanoemulgel could promote and accelerate the repair of buccal traumatic ulcers.

**Methods:**

In total, 34 Wistar rats were randomized into two groups, where one group was treated with Aloclair Plus Gel® (positive control) and the other received the green tea extract nanoemulgel. A wound with a diameter of 3 mm was created in the buccal mucosa using a biopsy punch. As much as 20 μL of the nanoemulgel was applied to the wound once daily for 4 days. Tissue samples were collected by sacrificing the rats on days 1, 3, 5, 7, and 14, and embedding the samples in paraffin. The nanoemulgel was also applied to the skin on the back of the rats to determine allergic reactions. Allergic reactions, wound diameters, perfusion, and COX-2 expression levels were evaluated. Allergic reactions were assessed clinically and histologically. Analysis of variance was used to assess differences in the wound diameter, perfusion patterns, and COX-2 expression, and Tukey's test to assess differences between groups.

**Results:**

The green tea extract nanoemulgel was tolerated well with no signs of allergy. Significant increases in perfusion were found on day 5 and COX-2 expression peaked on day 3, with progressive wound contraction.

**Conclusion:**

Our findings showed that the green tea extract nanoemulgel formulation accelerated the repair of traumatic buccal mucosal ulcers through enhanced neovascularization, inflammatory response modulation, and wound diameter reduction.

## الملخص

**أهداف البحث:**

هدفت الدراسة إلى تقييم ما إذا كان نانوإيمُلجل مستخلص الشاي الأخضر قادرًا على تعزيز وتسريع ترميم القرح الرضّية في مخاطية الخد.

**طرق البحث:**

شملت الدراسة 34 جرذًا من سلالة ويستار، وجرى توزيعها عشوائيًا إلى مجموعتين؛ عولجت إحدى المجموعتين بـ Aloclair Plus Gel بوصفه ضابطًا إيجابيًا، بينما تلقت المجموعة الأخرى نانوإيمُلجل مستخلص الشاي الأخضر. أُحدث جرح بقطر 3 مم في مخاطية الخد باستخدام أداة خزعة دائرية. وطُبِّق مقدار 20 ميكرولتر من النانوإيمُلجل على الجرح مرة واحدة يوميًا لمدة أربعة أيام. جُمعت العينات النسيجية بعد التضحية بالحيوانات في الأيام الأول والثالث والخامس والسابع والرابع عشر، ثم ضُمِّنت في البارافين. كما طُبِّق النانوإيمُلجل على جلد ظهر الجرذان للتحقق من حدوث تفاعلات تحسسية. وتم تقييم التفاعلات التحسسية، وأقطار الجروح، والتروية الدموية، ومستويات تعبير إنزيم الأكسدة الحلقية-2. وقُيِّمت التفاعلات التحسسية سريريًا ونسيجيًا. واستُخدم تحليل التباين لتقييم الفروق في قطر الجرح، وأنماط التروية، وتعبير إنزيم الأكسدة الحلقية-2، كما استُخدم اختبار توكي لتحديد الفروق بين المجموعات.

**النتائج:**

أظهر نانوإيمُلجل مستخلص الشاي الأخضر تحمّلًا جيدًا دون وجود أي دلائل على حدوث تفاعل تحسسي. ولوحظت زيادات معنوية في التروية الدموية في اليوم الخامس، بينما بلغ تعبير إنزيم الأكسدة الحلقية-2 ذروته في اليوم الثالث، مع حدوث تقلّص تدريجي في الجرح.

**الاستنتاجات:**

أظهرت نتائجنا أن تركيبة نانوإيمُلجل مستخلص الشاي الأخضر أسهمت في تسريع ترميم القرح الرضّية في مخاطية الخد من خلال تعزيز تكوّن الأوعية الدموية الجديدة، وتعديل الاستجابة الالتهابية، وتقليل قطر الجرح.

## Introduction

Traumatic ulcers are oral mucosal lesions caused by mechanical, thermal, chemical, or electrical trauma, with an estimated prevalence of 83.6%.^1^ Deep lesions present as white or red areas of epithelial loss, often with a yellow fibrinous exudate centrally and an erythematous border, where they are rounded in shape and variable in size.^2^ Wounding of the oral mucosa induces COX-2 expression in the basal epidermal layer, fibroblasts, and peri-epithelial capillaries, and COX-2 catalyzes the synthesis of inflammatory mediators including thromboxane and prostaglandins. The presence of ulcerative lesions in acute or chronic conditions, which are manifestations of wounds in the oral cavity, can trigger the wound healing process.^3^, ^4^, ^5^, ^6^

Oral mucosal wound healing differs from cutaneous repair in terms of anatomical and microenvironmental differences. Saliva maintains hydration and a buffered pH of about 5.5–7, promoting more rapid repair with minimal fibrosis. Healing is a continuous, overlapping sequence involving hemostasis, inflammation, proliferation, and maturation, and the inflammatory phase is critical for containing the insult and clearing the wound to enable prompt progression to proliferation. The wound healing process requires perfusion to ensure oxygen and nutrient availability at the wound site, facilitate immune cell infiltration, and remove waste products and exudate, thereby centrally controlling the rate and quality of the healing process.^6^^,^^7^

Oral mucosal wound healing can be accelerated with herbal remedies, which have minimal side effects compared with synthetic drugs. Herbal remedies with therapeutic effects that accelerate wound healing are derived from natural ingredients, such as green tea (*Camellia sinensis*). The fresh, young leaves of the green tea plant are utilized for this purpose because they retain more active substances and vitamins than other parts. Fresh, young green tea leaves contain catechins, where the main component is epigallocatechin 3-gallate (EGCG), which comprises approximately 60% of the total catechin content. EGCG has been shown to act as an anti-inflammatory agent in vivo by suppressing the synthesis of inflammatory mediators in oral mucosal wounds, thereby accelerating wound healing.^8^, ^9^, ^10^, ^11^

The effectiveness of herbal remedies is still limited by the low bioavailability, solubility, and stability of the active ingredients in the oral mucosa. The active ingredients in herbal remedies are often degraded by saliva and it is difficult for them to penetrate the mucosal layer due to their large molecular size. Nanotechnology is being developed to improve the stability and solubility of herbal remedies. A nanoemulgel is a combination of a nanoemulsion with a hydrogel-based gel system that it is expected to increase the stability, viscosity, and contact time of the active substance on the buccal mucosa.^12^^,^^13^

In the present study, we assessed whether a *Camellia sinensis* (green tea) extract nanoemulgel could improve the repair of traumatic buccal ulcers by measuring allergic reactions, wound size, blood perfusion, and COX-2 expression levels. The novel aspect of this study was the simultaneous measurement of allergic reactions, wound diameter, local perfusion, and COX-2 expression, providing a coherent, multi-level data set. The results demonstrate the ability of the nanoemulgel to overcome the limitations in terms of the bioavailability, solubility, and stability of the active ingredients of tea extract in the buccal mucosa.

## Materials and Methods

This study was approved by the Research Ethics Committee, Faculty of Dentistry, Prof. Soedomo Dental Hospital, Gadjah Mada University (letter no. 65/UN1/KEP/FKG-RSGM/EC/2025). Fresh tea leaves were supplied by PT Pagilaran Indonesia under letter no. 009/D.a/Dir/IV/2025. The Plant Systematics Laboratory, Faculty of Biology, Universitas Gadjah Mada, identified the leaves as *Camellia sinensis* (L.) Kuntze. Materials and the tea extract nanoemulgel were produced at Surabaya Academy of Pharmacy (letter no. 4185/UN1/KG.1/PT/2025). COX-2 antibody was purchased from Novus Biologicals, LLC, USA. Immunohistochemistry kits were purchased from Elabscience, Texas, USA. Chemical materials used for hematoxylin–eosin staining and other chemicals were obtained from the Integrated Research Laboratory, Faculty of Dentistry, Universitas Gadjah Mada. The research subjects were 34 Wistar rats obtained from the Faculty of Biology, Universitas Gadjah Mada.

### Production of tea extract and nanoemulgel

Fresh tea leaves were separated from the stems and sliced. The tea leaves were dried in the sun and blended. The procedure continued by sifting the tea leaves and weighing them. Next, 96% alcohol was added to the green tea powder, before allowing the mixture to stand for 3 days. The tea leaf maceration process was followed by three filtration steps. The filtered material was evaporated at 50 °C, subjected to a pressure of 200 mbar, and stirred at 40 rpm. Manufacture of the green tea extract nanoemulgel, organoleptic testing, and particle size analyzer measurements were conducted at the Academy Pharmacy, Surabaya-Indonesia. The tea leaf extract nanoemulgel with a concentration of 10% was prepared according to the protocol specified by the Academy Pharmacy, Surabaya-Indonesia.

The green tea leaf extract was mixed with propylene glycol in a mortar and ground until homogeneous, before adding PEG-40 HCO and Tween-80 sequentially, grinding to uniformity, adding 96% ethanol, and mixing to form the oil phase. Nipagin was dissolved in half the distilled water to form the aqueous phase. The aqueous and oil phases were combined with a stirrer at 1000 rpm and 50 °C for 10 min to form a nanoemulsion. The gel was prepared by sprinkling sodium carboxymethyl cellulose evenly onto the heated residue in distilled water, before allowing to hydrate for 15 min and mixing until a homogeneous gel formed. The nanoemulsion was gradually incorporated into the gel under stirring until a uniform mixture was obtained, before reducing the particle size using an Ultra-Turrax at 15,500 rpm (scale 4) for 10 min (with short cycles to avoid overheating). Any entrapped foam was removed by centrifugation for 10 min at 1300 rpm (scale 3), before collecting the final green tea nanoemulgel for characterization and storage.

### Formation of traumatic ulcers in the buccal mucosa

In total, 34 Wistar rats were randomly assigned to either the treatment or control group. Each rat was sedated with ketamine (0.1 mL/100 g, intramuscular), before using a 3 mm biopsy punch to create an ulcer on the cheek mucosa. The nanoemulgel was applied to the treatment group and the control group received Aloclair Plus®. Therapy was administered once daily for 4 days using a micropipette with as much as 20 μL per day. Anesthesia was administered to the experimental animals on days 1, 3, 5, 7, and 14 after treatment. Wistar rats were decapitated after intramuscular administration of a lethal dose of ketamine at twice the anesthetic dose (anesthesia dose of 0.2 mL/100 g body weight) in accordance with ethical guidelines for minimizing pain during death. On each of days 1, 3, 5, 7, and 14, three rats were sacrificed from each group and the ulcer area on the buccal mucosa was biopsied. Tissue slices with a thickness of about 2–3 mm were fixed in 10% buffered formalin for 24 h, and then processed for paraffin embedding.

### Perfusion measurement using laser speckle contrast imaging microscopy

Laser speckle contrast imaging was used to measure changes in the speckle pattern produced when coherent light (laser) was scattered by tissue containing moving particles (red blood cells). Time variations in the speckle intensity were converted into spatial or temporal contrast, which correlated with the relative velocity of blood flow and tissue perfusion. Food residues surrounding the buccal ulcers were cleared from anesthetized Wistar rats, and a headrest was used to stabilize the animals and prevent motion. The ulcer area on the buccal mucosa was marked as the region of interest and images were then captured. Perfusion was observed on days 1, 3, 5, 7, and 14 after treatment.

### Allergic reaction testing on skin on the backs of rats

Wistar rats were acclimatized for ≥7 days per group (n = 4) and the fur was shaved from their backs. Tea extract nanoemulgel and Aloclair Plus® were applied to the skin on the back of the rats, before covering with gauze. Clinical observations were made 24, 48, 72, and 96 h after application. At 96 h, the rats were lethally anesthetized and decapitated. The skin from the backs of the rats was fixed in 10% buffered formalin and processed to prepare paraffin blocks.

### Histological preparation

Tissues were fixed for 1 day in 10% neutral buffered formalin to stabilize the cells and prevent decay. After drying, each sample was trimmed to a thickness of 0.3–0.5 mm with a scalpel, placed in a cassette and basket, and processed in an automated tissue processor for a cycle of 20 h. The tissue was dehydrated to remove the water content, facilitating subsequent sectioning. The dehydration process was conducted in stages by immersing the tissue in the following alcohol concentrations: 70% alcohol (1.5 h), 80% alcohol (1.5 h), 95% alcohol I (1 h), 95% alcohol II (1 h), absolute alcohol I (1 h), absolute alcohol II (1.5 h), and absolute alcohol III (2 h). A clearing stage was then performed to remove any residual alcohol from the tissue using xylene I (1.5 h) and xylene II (2 h). The final stage involved paraffin infiltration to fill the cavities within the tissue with liquid paraffin at a temperature of 57–59 °C. The paraffin infiltration process was performed in stages, starting with immersion in liquid paraffin I for 1.5 h, followed by immersion in liquid paraffin II for 2 h. The next stage was the embedding process. The paraffin block was cut by using a microtome to a thickness of 3 μm. The ribbon-shaped tissue pieces were then placed in a water bath at 50 °C, removed on a slide, drained, and labeled with an identification number using a pencil. The slide was then incubated on a hot plate at 58 °C for 15–30 min to evaporate any remaining water, allowing the tissue to adhere firmly to the slide in preparation for staining.

### Hematoxylin–eosin staining

Hematoxylin–eosin staining commenced by immersing the slide in xylene three times each for 3 min to dissolve and remove any remaining paraffin. Next, the slide was immersed in alcohol solutions with decreasing concentrations (100%, 95%, 80%, and 70%) each for 2 min, which gradually reintroduced water into the tissue, allowing the tissue structure to return to its original state within the experimental animal. The slide was then immersed in running water for 4 min to remove any remaining alcohol. Cell nuclei were stained a purplish-blue color by immersing the slide in Mayer's hematoxylin. The slide was then immersed in eosin for 0.5–2 min to stain the cytoplasm, before washing in three containers of clean water, each with three dips, to remove any remaining eosin that was not absorbed by the tissue. After staining, the dehydration stage was performed by immersing the slide in a graded series of alcohol solutions (70%, 80%, 95%, and 100%) to remove the water content added during preparation. The process was continued by immersing two times in xylol for 2 min to remove any remaining alcohol, so the tissue appeared brighter and clearer. The final stage involved mounting and covering with a cover slip.

### Immunohistochemical staining

Slides were first deparaffinized by immersing in xylene (two changes, 5 min each), before passing through a graded series of alcohol concentrations to remove the paraffin: 100% (two changes, 3 min each), 95% (3 min), 80% (3 min), and 70% (3 min). After draining, the slides were placed in a humidified chamber lined with wet wipes to prevent them from drying out. Endogenous peroxidase was blocked by applying a drop of 3% H_2_O_2_ to each slide and incubating at room temperature for 10 min, followed by three washes in phosphate-buffered saline (PBS) for 2 min. To lower background staining, normal goat blocking buffer was applied and the slides were incubated for 30 min at 37 °C. Finally, 50 μL of rabbit polyclonal anti-COX-2 antibody (Novus Biologicals, USA) was added at a dilution of 1:100, before the slides were incubated overnight at 4 °C.

Following incubation with the antibody for 24 h, slides were washed in PBS three times (2 min each time). The reverse sides of the slides were dried with a tissue and polyperoxidase-anti-mouse/rabbit IgG (E-IR-R217B) was added, before incubating for 20 min at room temperature or 37 °C and then washing three times in PBS for 2 min each. Staining was performed by immersing the slides in 1:20 3,3′-diaminobenzidine (DAB) chromogen solution with the substrate for 5 min or until a tan or yellow-brown color appeared, indicating a positive signal, with monitoring to avoid overstaining. The slides were washed with distilled water to remove any remaining reagent. Counterstaining was performed by dropping Mayer's hematoxylin on a glass slide, incubating for 3 min at room temperature, and washing in distilled water to ensure that any residual hematoxylin was completely rinsed off. Dehydration was performed by immersing the slides in a graded series of alcohol solutions (70%, 80%, 95%, and 100%), and immersing two times in xylol for 2 min. The final stage involved cleaning the slides, adding a drop of mounting medium, and covering with a cover slip. The slides were dried for 24 h to ensure that the mounting medium completely hardened. Positive COX-2 was indicated by brown/DAB staining in the cytoplasm or perinuclear region of expressing cells.

## Results

### Phytochemical screening

Phytochemical screening showed that the tea extract contained flavonoids, saponins, tannins, terpenoids, steroids, and alkaloids. The total phenol content of the tea extract was 194.72 mg gallic acid equivalents (GAE)/g extract. The total flavonoid content was 191.67 mg quercetin equivalents (QE)/g extract. Both values were in the high category for plant extracts and in the range of ∼190–195 mg/g. These results indicate that the extract prepared using tea from PT Pagilaran Indonesia was very rich in phenolics and flavonoids. The almost equal phenol and flavonoid levels indicate that the majority of the phenolic fraction in the extract was rich in flavonoids (catechins, tea polyphenols).

### Stability of green tea extract nanoemulgel

The stability of the tea extract nanoemulgel was tested by storing the preparation at a temperature of 4 °C ± 1 °C for 24 h, followed by a temperature of 40 °C ± 1 °C for 24 h. Physical stability testing was conducted for 12 days, including analysis of organoleptic characteristics (color, odor, and shape), pH, spreadability, and homogeneity (Table 1).

**Table 1:** Organoleptic and visual homogeneity test results.

Evaluation	Results
Color	Brownish green
Odor	Characteristic tea aroma
Texture	Semi solid (Gel)
pH	5.48
Homogeneity	Homogenous
Spread ability	6 cm

### Fourier transform infrared (FTIR) spectroscopy results

**Figure 1:**
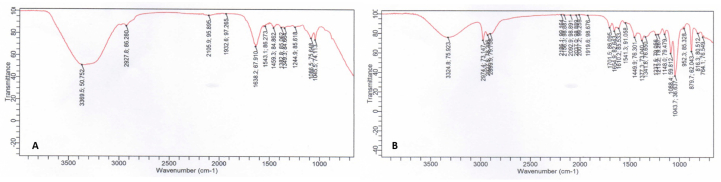
FTIR results for the base preparation (A) and tea extract nanoemulgel (B).

The FTIR results for the base preparation and tea extract nanoemulgel are shown in Figure 1.

Sample G1 (base) produced characteristic matrix bands (C–O, weak C=O, and aromatic/amide) and a weak OH band at ∼3369 cm^−1^. Sample G2 (nanoemulgel + tea extract/EGCG) produced additional and/or shifted peaks, with a clearer OH band (∼3325 cm^−1^), a more prominent C=O peak (∼1701 cm^−1^), and increases in the intensity and amount of peaks in the 1600–1500 cm^−1^ region (aromatic/amide).

The FTIR spectrum indicated the characteristic aromatic phenolic signature (EGCG/tea extract) and that for the nanoemulgel matrix (surfactant/ester/C–O), as well as possible hydrogen bonding/physical interactions between the active ingredient and carrier. The results demonstrated: (1) the presence of a broader/more intense OH band in G2 (∼3325 cm^−1^), supporting the inclusion of phenolic components (EGCG/tea extract) in the nanoemulgel and the possibility of hydrogen bonding between EGCG and the carrier matrix; (2) the emergence of a strong C=O peak at 1701 cm^−1^ in G2, which was not dominant in G1, indicating a new carbonyl component or a change in the carbonyl environment as an interaction with EGCG; and (3) the increase in the intensity in the 1610–1540 cm^−1^ region in G2 was consistent with an increase in the content of conjugated aromatic structures (catechins) and/or changes in the amide in the carrier material.

### Particle size analysis measurements

**Figure 2:**
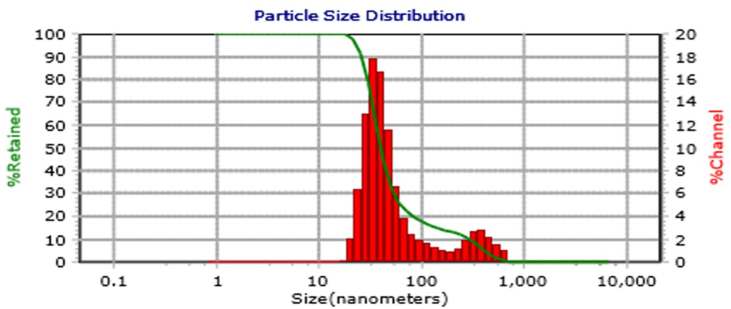
Particle sizes in tea extract nanoemulgel, with a median size (D50) of 282 nm.

The particle size analysis measurements are shown in Figure 2.

The particle sizes in the tea extract nanoemulgel were in the mid-nanometer to submicron range with a median (D50) of 282 nm, and most particles measured around ∼200–220 nm. The polydispersity index of 0.228 indicated a moderately narrow size distribution, and this value is generally acceptable for nanoemulgel formulations (values < 0.3 indicate relatively good polydispersity).

### Clinical observation of allergic reaction on skin on backs of rats

**Figure 3:**
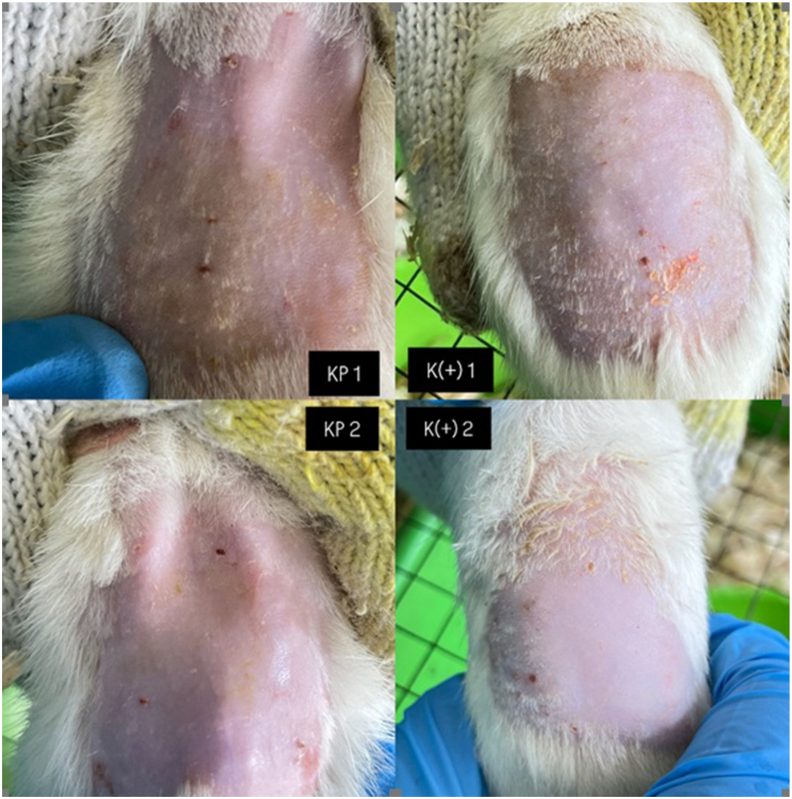
Clinical observations after application of green tea extract nanoemulgel and positive control for 4 days on skin on the backs of rats indicated no allergic reactions. KP1 and KP2: treatment group on day 1 and day 4, respectively. K(+)1 and K(+)2: positive control (Aloclair Plus®) group on day 1 and day 4, respectively.

Clinical observations after administration of the green tea extract nanoemulgel and positive control (Aloclair Plus®) to the skin on the backs of rats for 4 days indicated no allergic reactions. The allergic reaction test observations are shown in Figure 3.

**Figure 4:**
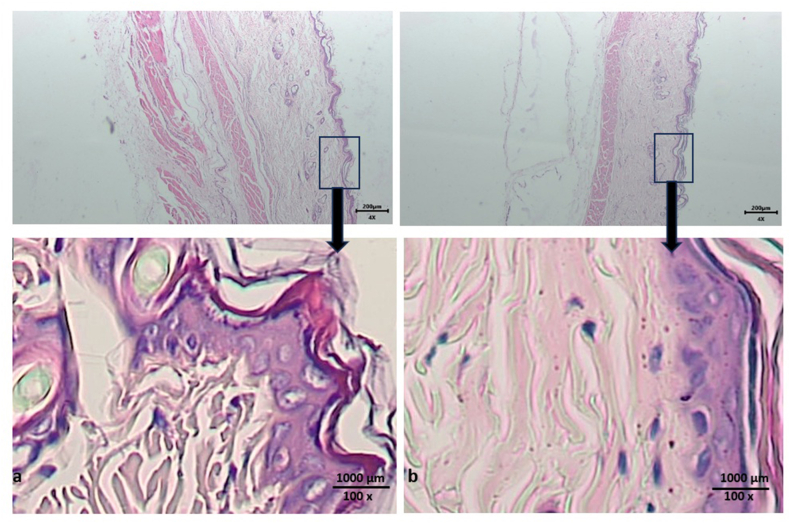
Histological observations after application of green tea extract nanoemulgel (A) and positive control (B) for 4 days on skin on the backs of mice indicated no allergic reaction and no damage to the epithelium or connective tissue. Hematoxylin–eosin staining at magnifications of 4× and 100×.

Hematoxylin–eosin staining of tissues from skin on the backs of rat after day 4 indicated no infiltration of inflammatory cells or mast cells in either the treatment or control groups (Figure 4).

### Wound diameters

**Figure 5:**
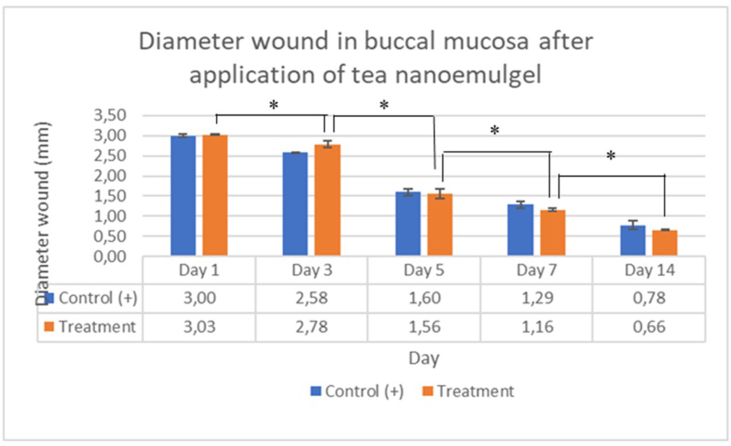
Wound diameters (mm) on buccal mucosa after application of green tea extract nanoemulgel, indicating a significant decrease (∗according to Tukey's test) over time. The diameters were smaller from day 5 in the treatment group compared with the control.

The wound diameters in the traumatic ulcer buccal mucosa after the application of green tea extract nanoemulgel and positive control are shown in Figure 5.

Wound diameter and perfusion data were then tested for normality using the Shapiro–Wilk test, and the result of *p*> 0.05 indicated a normally distributed data distribution. Homogeneity testing using Levene's test indicated homogeneity of variance (*p*> 0.05). The test results obtained by two-way analysis of variance (ANOVA) are shown in Table 2, Table 3.

**Table 2:** Two-way ANOVA test results to assess the effects of applying green tea extract nanoemulgel on the diameter of buccal mucosa wounds.

Tests of Between-Subjects Effects					
Dependent Variable: Effect of the nanoemulgel tea on diameter wound (mm)					
Source	Type III Sum of Squares	df	Mean Square	F	Sig.
Corrected model	15.210^a^	9	1.690	306.990	0.000
Intercept	67.822	1	67.822	12320.153	0.000
Group2	0.000	1	0.000	0.074	0.792
day2	15.139	4	3.785	687.504	0.000
Group2 ∗ day2	0.071	4	0.018	3.205	0.062
Error	0.055	10	0.006		
Total	83.087	20			
Corrected total	15.265	19			

a R Squared = 0.996 (Adjusted R Squared = 0.993).

**Table 3:** Two-way ANOVA test results to assess the effect of applying green tea extract nanoemulgel on perfusion in buccal mucosa injuries.

Tests of Between-Subjects Effects					
Dependent Variable: Effect of the nanoemulgel tea on perfusion (PU)					
Source	Type III Sum of Squares	df	Mean Square	F	Sig.
Corrected model	1382398.951^a^	9	153599.883	6.732	0.000
Intercept	60886426.128	1	60886426.128	2668.399	0.000
Group3	94720.583	1	94720.583	4.151	0.051
Day3	1193438.205	4	298359.551	13.076	0.000
Group3 ∗ Day3	94240.163	4	23560.041	1.033	0.407
Error	684527.584	30	22817.586		
Total	62953352.663	40			
Corrected total	2066926.535	39			

a R Squared = 0.669 (Adjusted R Squared = 0.569).

The two-way ANOVA results indicated a highly significant difference (*p*< 0.001) in the wound diameter after applying green tea extract nanoemulgel (Table 2). Comparison of the effects of each treatment according to the observation day (*p*< 0.05) indicated a significant decrease in wound diameter over time.

The results in Table 3 show that applying green tea extract nanoemulgel had a significant effect on perfusion, where perfusion changed significantly over the five observation days.

**Figure 6:**
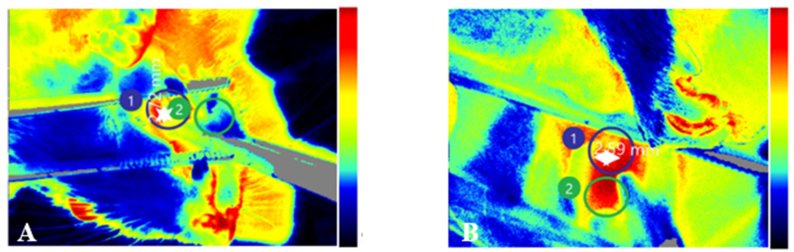
Observations of wound diameter and perfusion on day 3. The wound diameter was wider in the treatment group (A) than the control (B).

**Figure 7:**
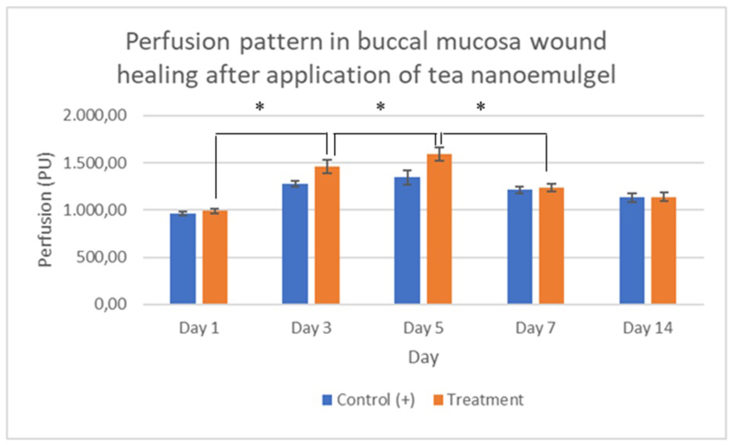
Perfusion was highest on day 5. The perfusion pattern (perfusion unit/PU) in the buccal mucosa wound healing process after applying green tea extract nanoemulgel indicated a significant increase in perfusion on day 3, and a peak was reached on day 5 (∗) in both the control and treatment groups, before then decreasing with time.

Statistical analysis was conducted using Tukey's test to determine differences between groups over time. The Tukey's test results indicated that the wound diameter decreased significantly over the observation period (Figure 6). Perfusion analysis indicated a significant increase starting on day 3 and peaking on day 5 (Figure 7).

**Figure 8:**
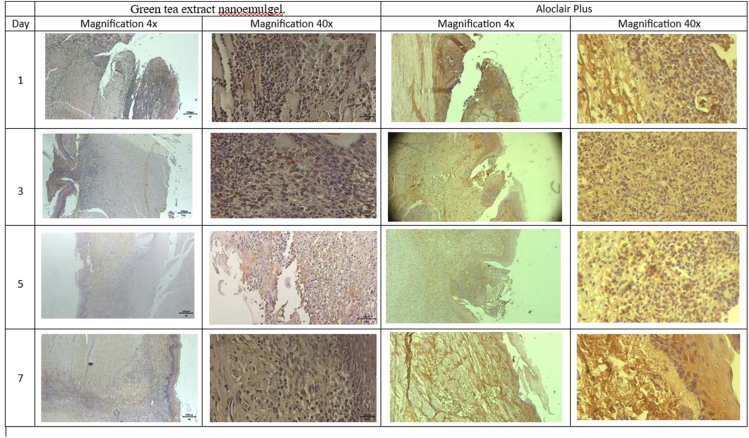
COX-2 expression shown by brown color in inflammatory cells. Very high expression was observed in wounds on day 3 after treatment with green tea extract nanoemulgel and Aloclair Plus.

The cell populations identified as COX-2 positive (Figure 8) in the wound area were mucosal basal epithelial cells, fibroblasts in the lamina propria/granulation tissue, periepithelial capillary endothelial cells, and inflammatory cells (e.g., macrophages and neutrophils).

**Figure 9:**
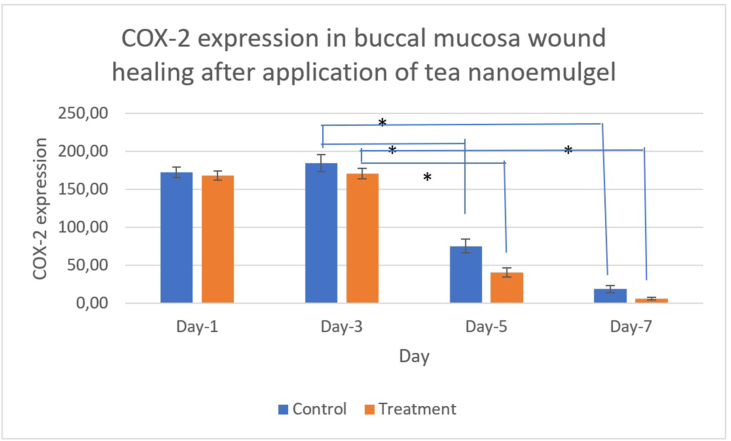
COX-2 expression decreased over time in both groups and was consistently lower under the tea nanoemulgel treatment than the control at every time point.

The data were normally distributed and homogeneous according to data normality testing using the Shapiro–Wilk test (*p*= 0.897) and homogeneity of variance analysis using Levene's test (*p*= 0.196). Furthermore, Tukey's test showed that COX-2 expression differed significantly between the tea nanoemulgel treatment group and control group on day 3 compared with days 5 and 7 (*p*< 0.05), but the differences were not significant on days 1 and 3 (Figure 9).

## Discussion

Phytochemical screening detected flavonoids, saponins, tannins, terpenoids, steroids, and alkaloids in the extract from tea provided by PT Pagilaran Indonesia. The total phenolic content was 194.72 mg GAE/g extract and the total flavonoid content was 191.67 mg QE/g extract, and both values were classified as high. The similar contents for the total phenols and total flavonoids indicate that flavonoids constituted a large proportion of the extractable phenolic pool. The observed profile is consistent with tea chemistry, where catechins and other tea polyphenols are dominant contributors to both phenol and flavonoid assays. The presence of saponins, tannins, terpenoids, steroids, and alkaloids showed that the phytochemical matrix was complex, and they may act synergistically with flavonoids.

The measured D50 value of 282 nm and wide tail indicated that the particles in the tea extract nanoemulgel formulation were submicron in size. This size could be suitable for use in local buccal mucosal therapy due to its potentially good retention. However, reducing the size to <200 nm is still necessary to improve the transmucosal absorption or intracellular delivery of EGCG. After topical application to skin on the backs of rat for 4 days (Figure 3), the green tea extract nanoemulgel group and positive control group treated with Aloclair Plus® showed no signs of allergic reactions. The clinical parameters monitored included absence of erythema, edema, or pruritus. The absence of local allergic signs suggests that the nanoemulgel matrix and the incorporated green tea extract did not induce an immediate or delayed type of hypersensitivity under the test conditions. Nanoemulgel carriers often allow controlled release and reduce direct contact with irritants by encapsulating bioactive molecules, which may reduce the local potential for irritation. The results obtained in the present study support previous findings that the nanoemulgel delivery system provides controlled release and local retention of lipophilic active ingredients, which could reduce the direct exposure of skin cells to high concentrations, thereby decreasing the potential for irritation. Encapsulation in a nanoemulgel can moderate the contact between bioactive molecules and the epidermis to reduce the immediate irritant response.^14^

The tea extract nanoemulgel prepared in this study contained high levels of flavonoids and tannins. These compounds are considered to have anti-inflammatory and antimicrobial activities. In addition, green tea polyphenolic components, particularly epigallocatechin 3-gallate, have anti-inflammatory properties that may attenuate the local inflammatory response and contribute to a lack of observable irritation or erythema after topical application. Previous studies have also shown that green tea extracts contains high levels of catechins, flavonoids, and tannins. The phytochemical components in tea leaves have anti-inflammatory and antimicrobial activities, which could reduce local inflammatory signals and microbial contributions to irritation during topical application. EGCG in tea leaves can modulate inflammatory pathways, such as NF-κB, inhibit proinflammatory cytokine production, and confer tissue-protective effects by reducing erythema and leukocyte recruitment.^15^

Aloclair Plus® is formulated as a soothing oral mucosal gel with established tolerability, and it was used as a positive control in the present study. The similar clinical outcomes obtained in the positive control and treatment groups indicated that the nanoemulgel's local safety profile was at least comparable to that of an accepted topical formulation. Aloclair Plus is made from aloe vera, sodium hyaluronate, glycyrrhetinic acid, and polyvinylpyrrolidone, all of which are considered to have anti-inflammatory properties.^16^

In this study, we limited topical application to the first 4 days to specifically target the inflammatory phase, which predominates in the initial 3–5 days after injury. This design tested whether early anti-inflammatory modulation produced sustained improvements in healing while minimizing potential interference with proliferative and remodeling processes.

Topical therapy was administered once daily for four consecutive days to concentrate exposure during the early inflammatory and proliferative phases of mucosal wound healing while minimizing repeated anesthesia and handling stress. The nanoemulgel carrier was selected because of its mucoadhesive and controlled release properties, which prolong local residence and maintain therapeutic concentrations after a single daily application; therefore, a short dosing course is expected to produce sustained local pharmacodynamic effects that can be assessed at later time points (days 1, 3, 5, 7, and 14). This design balanced ethical considerations, practical feasibility, and the biological timing of COX-2 modulation and angiogenesis, which drive wound contraction.

The average wound diameter (Figure 5, Figure 6) decreased in both groups (positive control and treatment) from day 1 to day 14, indicating that the healing process occurred under both conditions. The wound diameter was consistently smaller in the treatment group than the control group at almost all observation times. In the early phase (days 1–3), more rapid wound reduction was observed in the treatment group by day 3 than the control, indicating the early action of the green tea nanoemulgel in shortening the late inflammatory phase and promoting the transition to proliferation. During the proliferation phase (days 3–7), the statistically significant differences on days 5 and 7 further supported accelerated wound contraction and granulation tissue development using the green tea formulation. In the remodeling phase (days 7–14), on day 14, further shrinkage was observed in both groups until wound closure and tissue healing.

The mechanism associated with wound closure after applying the tea extract nanoemulgel could be explained by: (1) the anti-inflammatory properties of tea catechins, such as EGCG, possibly reducing the expression of proinflammatory cytokines and decreasing neutrophil infiltration, thereby accelerating the transition from the inflammatory phase to proliferation; (2) the antioxidant activities of tea components protecting epithelial cells and fibroblasts from oxidative damage to support cell proliferation and migration; (3) the antimicrobial effects of tea components including flavonoids/tannins reducing the presence of microbes as inhibitors of healing; and (4) the use of a nanoemulgel as a carrier of tea components could have resulted in nanoparticles increasing local penetration and retention, as well as allowing controlled release and longer exposure to the active components, thereby strengthening the initial therapeutic effect. This wound healing mechanism following tea nanoemulgel application is supported by previous research, which showed that tea extract had anti-inflammatory effects and EGCG acted as an antioxidant in wound healing. The application of tea catechins has a protective effect against reactive oxygen species (ROS) to accelerate wound healing. Tea flavonoids may also have antimicrobial properties that support wound closure.^17^, ^18^, ^19^, ^20^ In addition, previous studies indicated that tea extract gels could reduce the side effects of dental bleaching by reducing the number of inflammatory cells (neutrophils and macrophages) and increasing the number of reparative cells (odontoblasts) after extracoronal bleaching.^21^, ^22^, ^23^

Tea extract nanoemulgels are known to be effective carriers of nanoparticles that enhance local penetration and retention, as well as allowing controlled release and longer exposure to the active components of tea leaves, thereby strengthening the initial therapeutic effect. In the present study, the wound diameter decreased faster following application of the tea extract nanoemulgel compared with the control. Similarly, previous studies demonstrated the effectiveness of nanoemulgels as carriers for treating buccal mucosal traumatic ulcers following the application of pineapple stem extract and stem cells from human exfoliated deciduous conditioned medium (SHED-CM).^24^, ^25^, ^26^

The wound healing process requires perfusion to ensure the availability of oxygen and nutrients at the wound site, facilitate immune cell infiltration, and remove waste products and exudate, thereby centrally controlling the rate and quality of the healing process. Perfusion plays a role in every phase of wound healing. Perfusion decreases during the hemostasis phase, before increasing in the inflammatory phase leading up to the proliferation phase. Perfusion stabilizes during the remodeling phase. Figure 7 showed that buccal mucosa wound perfusion increased in the treatment group compared with the control group, where the differences were significant on days 3 and 5, indicating that applying the tea extract nanoemulgel increased local blood flow in the early to proliferative phases of wound healing. The results obtained in the present study suggested an increase in early angiogenesis. Peak perfusion occurred on days 3–5, which is consistent with the activation of angiogenesis and the formation of new capillaries to support the supply of oxygen and nutrients during the proliferative phase. The mechanism that allowed the tea nanoemulgel to increase perfusion during buccal mucosa wound healing may be explained as follows: (1) the nanoemulgel containing tea components or their bioactive factors could have increased the expression or bioactivity of proangiogenic factors, such as VEGF and TGF-β, thereby accelerating the recruitment and proliferation of endothelial cells; (2) the anti-inflammatory effects of the tea extract nanoemulgel may have modulated the microcirculation, where the reduction of local inflammation by catechins, including EGCG components, could have reduced edema and microvascular stagnation, thereby restoring effective perfusion and facilitating the transition to the tissue formation phase; and (3) the nanoemulsion formulation could have affected the bioavailability of tea extract active components, where the nanoscale size and gel matrix enhanced local retention and penetration, allowing the controlled release of active components to maintain proangiogenic stimuli longer than the control. The results obtained in the present study agree with previous research, which showed that a tea extract gel could affect the number of blood vessels in dental pulp after extracoronal bleaching using 40% hydrogen peroxide.^27^ Our results are also supported by previous findings that a topical nanoemulgel accelerated re-epithelialization, granulation tissue formation, and angiogenesis in wound models, and thus it could have increased perfusion and angiogenesis in the early to proliferative phases.^28^ The nanoemulgel formulation could have increased the signs of vascularization and generation of new capillaries in the treated tissue compared with the control through proangiogenic mechanisms characterized by increased expression of growth factors, such as VEGF and TGF-β, and the recruitment of endothelial cells.^28^

Figure 8, Figure 9 show how the COX-2 levels in the treatment group varied over time compared with the control. The COX-2 levels were similar on day 1, but increased in both groups by day 3 with a slightly higher level in the treatment group. They then decreased markedly by day 5 in the treatment group compared with the control, followed by low levels on day 7 with a slightly lower level in the treatment group. The significant differences found between the groups and across time points were consistent with the accelerated resolution of COX-2 mediated inflammation in the treatment group. This result indicates that COX-2 upregulation in the early post-injury period reflected an acute inflammatory response that supported hemostasis and early cell recruitment. The peak in COX-2 on day 3 was compatible with active inflammatory signaling preceding angiogenic and proliferative events. The pronounced reduction in COX-2 on day 5 in the treatment group indicated earlier attenuation of inflammation and a shift toward the proliferative phase compared with the control. The lower COX-2 level in the treatment group by day 7 indicated the resolution of inflammation and progression to tissue remodeling. The results obtained in the present study agree with previous research, which showed that a tea extract gel reduced inflammation according to the decrease in COX-2 expression in dental pulp after extra-coronal bleaching using 40% hydrogen peroxide.^29^

The effects of the tea extract nanoemulgel on COX-2 expression, perfusion, and angiogenesis may be explained by the following mechanisms: (1) modulation of inflammation and COX-2, where tea-derived catechins in the nanoemulgel, especially EGCG, had anti-inflammatory effects that downregulated inducible inflammatory enzymes including COX-2, thereby accelerating the transition from inflammation to proliferation and reducing prolonged inflammatory vascular permeability; (2) enhanced proangiogenic signaling and endothelial activation, where the improved early perfusion and peak blood flow on days 3–5 could be explained by the combined actions of reductions in edema and restored microcirculation plus upregulation or the increased bioactivities of proangiogenic mediators, such as VEGF and TGF-β, promoting endothelial cell recruitment and capillary sprouting; and (3) the formulation increasing bioavailability and sustaining local exposure, where the nanoscale droplets and gel matrix increased the local retention, tissue penetration, and controlled release of bioactive tea components to maintain a proangiogenic stimulus during the critical early proliferative window while also avoiding prolonged proinflammatory signaling.^30^, ^31^, ^32^

The COX-2 pattern that supported perfusion and angiogenesis was demonstrated by the increase in the COX-2 level on day 3, which coincided with the initiation of angiogenic signaling and increased perfusion. The perfusion peak on days 3–5 coincided with endothelial proliferation and the formation of new capillaries that supplied oxygen and nutrients to support granulation. The more rapid downregulation of COX-2 in the treatment group on day 5 reduced inflammatory edema and microvascular stasis, increasing the effective microcirculatory flow even as new functional capillaries formed. The latter effect was characterized by an earlier and stronger angiogenic response, followed by more rapid resolution, consistent with increased early angiogenesis and improved wound perfusion.

The results obtained in the present study demonstrate that early increased perfusion and controlled COX-2 modulation can support tissue oxygenation, nutrient delivery, and accelerated granulation, predicting faster re-epithelialization and improved healing quality. The tea extract nanoemulgel had a balancing effect on the anti-inflammatory potential and proangiogenic signaling, which was crucial for avoiding the excessive suppression of angiogenesis or prolonged inflammation. Furthermore, applying the tea extract nanoemulgel formulation at an appropriate dose maintained its proangiogenic effect on days 3–5 while allowing timely resolution of COX-2 induced inflammation. These results agree with previous reports that nanoemulgel formulations are effective as carriers of active substances from pineapple stem extract and SHED-CM, which were shown to be useful for accelerating the healing of oral mucosal wounds.^24^^,^^25^

EGCG and related green tea catechins have been shown to downregulate COX-2 expression and reduce PGE_2_ production in multiple cell types, where they act by suppressing NF-κB and MAPK signaling. In the present buccal ulcer model, the tea extract nanoemulgel produced an early COX-2 peak (day 3), followed by a more rapid decline compared with the control, together with increased early perfusion and accelerated wound contraction. This pattern is more consistent with the temporal modulation of COX-2 activity levels rather than continuous inhibition. COX-2 derived PGE_2_ has both proangiogenic and proinflammatory roles, so the transient, localized attenuation of excessive COX-2/PGE_2_ signaling is likely to promote resolution and repair while preserving the early angiogenic signals required for healing. Future studies should quantify tissue PGE_2_ levels, COX-2 enzymatic activity levels, and downstream angiogenic mediators (e.g., VEGF) to confirm the mechanistic link between EGCG delivery from the nanoemulgel and the observed histological and perfusion changes.

The results obtained in this study are useful for clinical development and support the improvement of public health in accordance with Sustainable Development Goal (SDG) 3 (Good Health and Well-being) as follows: (1) better oral mucosal wound healing reduces pain, risk of infection, and functional recovery time, supporting individual health and quality of life; (2) topical formulations (tea extract nanoemulgel) that work locally and are safe and affordable can expand access to effective wound care in primary and community health services in line with SDG 3 targets to reduce morbidity from preventable conditions and ensure access to quality essential health services; and (3) documented safety (no allergic reactions) and proven acceleration of healing support scale-up studies and integration into oral health promotion programs.

A limitation of the present safety assessment was the small sample size used for the initial allergic reaction screening. Clinical or histologic signs of irritation were not observed in these animals, but the cohort was too small to exclude less frequent or delayed hypersensitivity reactions. To address this issue, we will perform follow-up tests that include a vehicle (blank nanoemulgel) control and expanded group sizes (n = 4–6 per group), as well as in vitro irritation and sensitization assays. These additional studies will provide the statistical power and relevant regulatory data needed to characterize the local safety profile of the formulation prior to clinical translation.

## Conclusion

The green tea nanoemulgel was non-allergenic and accelerated repair by rapidly modulating inflammation and promoting angiogenesis, as shown by the COX-2 peak on day 3 followed by a significant decline on day 5, which paralleled increased perfusion. These effects indicate that the tea extract nanoemulgel accelerated the transition from the inflammatory phase to the proliferative phase, improved the local microcirculation, and may have supported tissue repair.

## Ethical approval

This study was approved by the Research Ethics Committee, Faculty of Dentistry, Prof. Soedomo Dental Hospital, Gadjah Mada University (letter no. 65/UN1/KEP/FKG-RSGM/EC/2025).

## Authors contributions

JH, IDS, and NAP conceived and designed the study, conducted research, provided research materials, and collected and organized data. JH, RTCT, and HS analyzed and interpreted data. JH, RS, DI, and PSAR wrote the initial and final drafts of the article. All authors have critically reviewed and approved the final draft and are responsible for the content and similarity index of the manuscript.

## Conflict of interest

The authors state they have no conflicts of interest concerning this study.
